# Search for antibodies against *Trichinella* in two synanthropic Procyonidae species from southeast Mexico: white-nosed coatis (*Nasua narica*) and raccoons (*Procyon lotor*)

**DOI:** 10.1007/s11259-023-10248-1

**Published:** 2023-11-08

**Authors:** Adrián Hernández-Ortiz, Emilio Rendón-Franco, Claudia-Irais Muñoz-García, Claudia Villanueva-García, Heriberto Caballero-Ortega, Jorge-Luis de-la-Rosa-Arana

**Affiliations:** 1 Facultad de Medicina Veterinaria y Zootecnia, Universidad Nacional Autónoma de México, Mexico City, México; 2 Department of Veterinary Microbiology, University of Saskatchewan, Saskatoon, Canada; 3 Departamento de Producción Agrícola y Animal, Universidad Autónoma Metropolitana, Mexico City, México; 4 Laboratorio de Ecología del Paisaje y Cambio Global, División Académica de Ciencias Biológicas, Universidad Juárez Autónoma de Tabasco, Villahermosa, México; 5 Laboratorio de Inmunología Experimental, Instituto Nacional de Pediatría, Mexico City, México; 6 Facultad de Estudios Superiores Cuautitlán, Universidad Nacional Autonoma de México, Cuautitlán Izcalli, México

**Keywords:** Trichinellosis, Immunosorbent assay (ELISA), Western blot (WB), Carnivore, Wildlife

## Abstract

*Trichinella* is a nematode that are spread by the consumption of parasitized meat. Carnivora, a mammalian order, serve as key hosts for this parasite. However, evidence of *Trichinella* in wildlife from the Neotropics is extremely scarce, with reports documenting its presence only for five carnivore species: two Felidae, one Otariidae and two Mustelidae. Other widely distributed species that are consumed as bushmeat, such as Procyonidae, have not been studied in this context. A long-term study was performed for antibodies against *Trichinella* in coatis (*Nasua narica*) and common raccoons (*Procyon lotor*) in southeastern Mexico. Between the summer of 2009 to the winter 2013, a total of 291 coati samples and 125 raccoon samples were collected from a tropical green area located within an urban zone. An Enzyme-linked immunosorbent assay (ELISA) was used to detect antibodies against the excretory and secretory products of *Trichinella spiralis* muscle larva. ELISA-positive samples were further confirmed by Western Blot analysis. Results showed no evidence of antibodies during the first two years of study. However, in 2011, a sudden appearance of anti-*Trichinella* occurred. The seroprevalence reached its highest peak of 43% for coatis during winter 2013 and 53% for raccoons in summer 2013. This is the first study that provides evidence of *Trichinella* circulation within a neotropical procyonid community.

**Supplementary Information:**

The online version contains supplementary material available at 10.1007/s11259-023-10248-1.

## Introduction

*Trichinella* is a genus of parasitic nematodes that are spread by the consumption of parasitized meat. Therefore, mammals of the order Carnivora play a crucial role in the life cycle of the parasite. They contribute to the persistence of the parasite in the wild environment and to a high prevalence (Pozio 2005). Additionally, for humans, it is a food-borne zoonosis. With the improvement on food safety coming from domestic animals, the importance of the consumption of infected meat from wildlife is growing (CDC 2019). In certain human populations, it is estimated that up to 8% of trichinellosis cases can be attributed to meat from wild carnivores (Dupouy-Camet 2014).

In the Americas, the consumption of meat from Carnivores is posed as a risk of trichinellosis. In Canada and the USA, most local cases of human trichinellosis have been linked to the consumption of game meat, primarily black bears (*Ursus americanus*), as has been the case in Argentina with puma (*Puma concolor*) (Dupouy-Camet 2014; CDC 2019; Ribicich et al. 2020). This situation could be particularly relevant in Latin American countries where bushmeat serves as an essential source of protein (Ávila Nájera et al. 2018).

Studies on *Trichinella* epidemiology have been done in over 80 carnivore species (Pozio 2005), but just few of them has been done in the tropical America (Neotropics), where *Trichinella* evidence is extremely scarce. For neotropical carnivores there are only case reports for puma (*Puma concolor),* guigna (*Leopardus guigna)*, lesser grison (*Galictis cuja*) and South American sea lion (*Otaria flavescens)* (Echeverry et al. 2021; Ribicich et al. 2020), as well as, the American mink (*Neovison vison),* one introduced mustelid to South America (Espinoza-Rojas et al. 2021).

The Family Procyonidae, within the Order Carnivora, comprises 17 species that are naturally distributed in the Americas. Some procyonids such as the common raccoons (*Procyon lotor*), the brown-nosed coatis (*Nasua nasua*) and the white-nosed coatis (*Nasua narica*) are synanthropic, commonly seen in urban areas (Alves-Costa 2004). Procyonids are frequently consumed as food sources by some human Latin American populations (Ávila Nájera et al. 2018). However, research on *Trichinella* in procyonids has been limited to the common raccoon, specifically within its North American (Nearctic) distribution (Smith et al. 1985), or in regions outside of the Americas where they are exotic fauna (Kobayashi et al. 2007). Despite their proximity to human settlements, neither of *Nasua* species have been examined for *Trichinella*.

Furthermore, most studies have been performed under cross-sectional sampling designs and were mainly focused on the study of a single target species (Zimmer et al. 2009). However, *Trichinella´s* natural parasitic dynamic must have long-term time variations and multiple host-parasite interactions. To elucidate *Trichinella* transmission, it is essential to carry out longitudinal studies of carnivores, particularly in those species that are used as food.

The aim of this study was a long-term follow-up study to search for antibodies against *Trichinella* in two synanthropic procyonidae species from southeast Mexico: white-nosed coatis and common raccoons.

## Materials and methods

The study was conducted at the urban park “Parque Museo de la Venta'', Villahermosa city, Tabasco state, Mexico (18°00′05.39’ N, 92°56′02.52’ W; 17masl). The climate is characterized by relative humidity about 80%, with an average annual temperature ranging from 22 to 28ºC. The park has 4.3 hectare of tropical disturbed forest surrounded by urban areas. The procyonids population in this park has previously been reported as 98 ± 26 for raccoons and 108 ± 8 for coatis (Martínez-Hernández et al. 2014).

Coatis and raccoons were captured twice a year (summer and winter) from 2009 to 2013, and since 2010 each animal was tattooed. Animals were captured as reported by Martínez-Hernández et al. (2014); afterwards, blood samples were taken and centrifuged to obtain serum and then stored at -20ºC until laboratory analysis. All animals were released at the capture site.

Female mice weighting approximately 20 to 25 ± 5 g, were orally infected with 23 *Trichinella spiralis* larvae (strain MSUS/ME/92/CM-92) per gram of body weight. The management of mice and experimental infection procedures were carried out in accordance with Mexican regulations (NORMA Oficial Mexicana NOM-062-ZOO-1999) and received ethical approval from the Coordination of Immunological Research at InDRE.

Muscular larvae were isolated from mice carcasses at 40 days post- infection using artificial digestion. Once the larvae were recovered, they were incubated in RPMI 1640 medium (Gibco BRL, Grand Island, NY) at 37 °C for 48 h in a humidified atmosphere, 95% air and 5% CO2. The excretory and secretory products (ESP) were released by the larvae into the medium and subsequently collected. To ensure the stability and integrity of the ESP, enzyme inhibitors were added to the recovered medium, which was subsequently utilized as the antigen for further analysis.

An in-House enzyme-linked Immunosorbent assay (ELISA) was performed as a screening test, and the Western blot (WB) as confirmatory test. Both tests were previously optimized by our research group for the diagnosis of trichinellosis (de-la-Rosa-Arana et al. 2021). To evidence the antigen–antibody reaction in the procyonid system, protein A peroxidase were used (Sigma-Aldrich St. Louis, MO, USA). The serological assays were validated with serum samples from mice experimentally infected with *Trichinella*.

Briefly, the best conditions for the ELISA were as follow, plates were coated with 100 µL/well of ESP (5 µg/mL) in carbonate buffer incubated overnight at 4 °C. The plate was washed 3 times with phosphate buffered saline (PBS) with 0.05% Tween 20 (PBS-T) and blocked with 1% bovine serum albumin (Euro-Clone, Milan, Italy) with PBS-T for 30 min at 37 °C. Then, 100 µL of each serum sample diluted 1:250 in PBS-T were added and incubated for 1 h at 37 °C. Protein A was diluted 1:2000 in PBS-T and added 100 µL/well for 1 h at 37 °C. The reaction was developed with 5 mg O-phenylendiamine (Sigma-Aldrich) citrate buffer solution and 4.5 µL 30% hydrogen peroxide. Reaction was stopped by adding 50 µL/well of 1N sulfuric acid. Samples were evaluated in duplicate; the optical density (OD) was obtained by a spectrophotometer at 490 nm wavelength. The cut-off point was calculated using the frequency distribution of OD values obtained with each one of the serum samples tested, according to Rendón-Franco et al. (2014) and with the mean of the low absorbance samples plus three times the standard deviation. Serum samples with absorbance values greater than the cut-off point were considered positive and were subjected to western blot to confirm the presence of antibodies against *Trichinella*. An equivalent number of negative samples was also tested.

The best conditions for WB were as follows, the ESP (100 µg) were separated in electrophoresis under reducing conditions on 12% polyacrylamide gels one hour at 200 V in an electrophoresis chamber (Bio-Rad Hercules, California 94,547, US). Proteins were transferred to nitrocellulose membranes (Bio-Rad) during 1 h at 100 V in a transfer system tank (Bio-Rad). Membranes were cut, and strips were blocked with 5% skim milk (Becton Dickinson and Company, Sparks MD, US) in PBS-T at 4ºC overnight. Strips were washed 3 times with PBS-T and 2 times with PBS; then each serum sample were diluted 1:100 in PBS-T and added (0.5 mL) to one strip and incubated in shaking during 1 h at room temperature. Protein A peroxidase was diluted 1:1000 in PBS-T and 0.5 mL were added to each strip and incubated as previously. The immunocomplexes were detected using 3,3’-diaminobenzidine (Sigma-Aldrich); reaction was stopped by washing with tap water.

Agreement between ELISA and WB was estimated by the Kappa index, and the test performance was estimated by the area under the curve (AUC) from a receiver operating characteristic curve (ROC). Seroprevalence and 95% Confidence intervals (CI) were calculated using Ausvet Epitools epidemiological calculators; afterwards, differences between species, season, sex, and age were analyzed using chi-square test with a level of significance at P < 0.05 using SPSS 21.0 software (IBM España, Santa Hortensia, Spain). Additionally, longitudinal individual seroconversion was analyzed for recapture animals.

## Results and Discussion

**Fig. 1:**
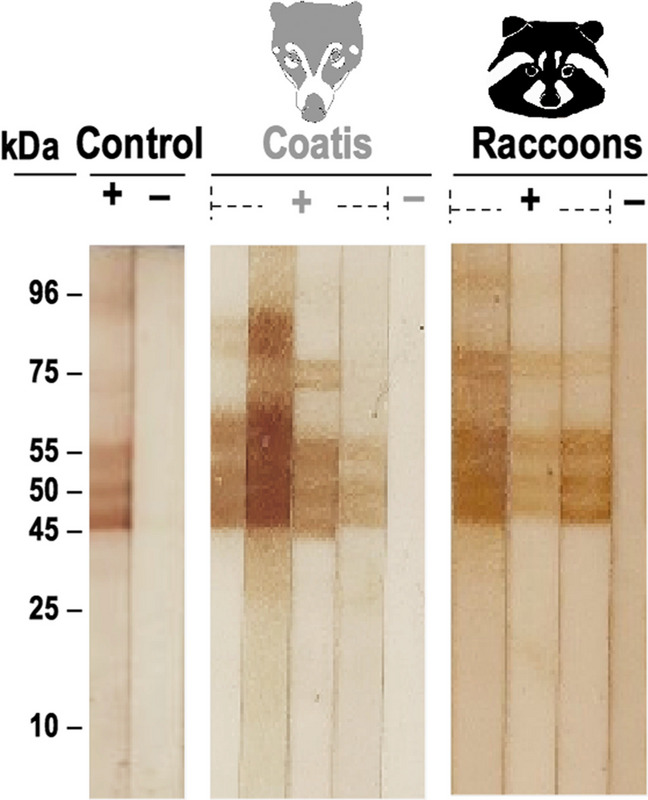
Western blot of serum samples of coatis and raccoons to the excretory secretory products of *Trichinella spiralis* muscle larvae

Sixty-nine coati serum samples and 50 raccoon serum samples were analyzed by WB and ELISA. All WB positive (n = 30 coatis; n = 27 raccoons) samples had three bands of 45, 50 and 55 kDa, characteristic of anti-*Trichinella* antibodies and some heavier additional bands (Fig. 1). For coati samples, sensitivity was 96.6%, specificity 84.6%, the ROC´s area under the curve (AUC) was 0.906 and Kappa value 0.797 (substantial agreement). For raccoon samples, sensitivity was 85.1%, specificity 86.9%, AUC was 0.843 and Kappa value 0.678 (substantial agreement).

All samples from 2009 were negative and since there was not individual information, these data were excluded from the analysis. A total of 241 serum samples of coatis and 105 of raccoons were collected between summer of 2010 and winter 2013. Overall seroprevalence was 18.2% for all procyonids. A higher seroprevalence was observed in raccoons (24.5%) than coatis (15.4%), with significant differences between species (p = 0.041). No differences were observed by season, sex, or age (Table 1).

**Table 1:** Seroprevalence for *Trichinella* in procyonids by categories. A, season of capture; B, sex of the animal; C, Age of the animal

A		Season	Positive	Total	Prevalence (%)	95% CI
Coati	Season	Winter	21	117	18	12.1–25.9
		Summer	16	124	12.9	8.1–19.9
	Total		37	241	15.4	11.4–20.4
Raccoon	Season	Winter	11	58	19	10.9–30.9
		Summer	15	48	31.3	20–45.3
	Total		26	106	24.5	17.3–33.5
Total	Season	Winter	32	175	18.3	13.3–24.7
		Summer	31	172	18	13–24.5
	Total		63	347	18.2	14.5–22.6
B		Sex	Positive	Total	Prevalence	95% CI
Coati		Female	20	131	15.3	10.1–22.4
		male	17	108	15.7	10.1–23.8
	Total		37	240		
Raccoon	Sex	Female	19	66	28.8	19.3–40.6
		male	7	40	17.5	8.8–32
	Total		26	106		
Total		Female	39	197	19.8	14.8–25.9
		male	24	148	16.2	11.2–23
	Total		63	346		
C		Age	Positive	Total	Prevalence	95% CI
Coati	Age	Adult	31	208	14.9	10.7–20.4
		Juvenile	6	32	18.6	8.9–35.3
	Total		37	240		
Raccoon	Age	Adult	22	89	24.7	16.9–34.6
		Juvenile	4	17	23.5	9.6–47.3
	Total		26	106		
Total	Age	Adult	53	297	17.9	13.9–22.6
		Juvenile	10	49	20.4	11.5–33.6
	Total		63	346		

**Fig. 2:**
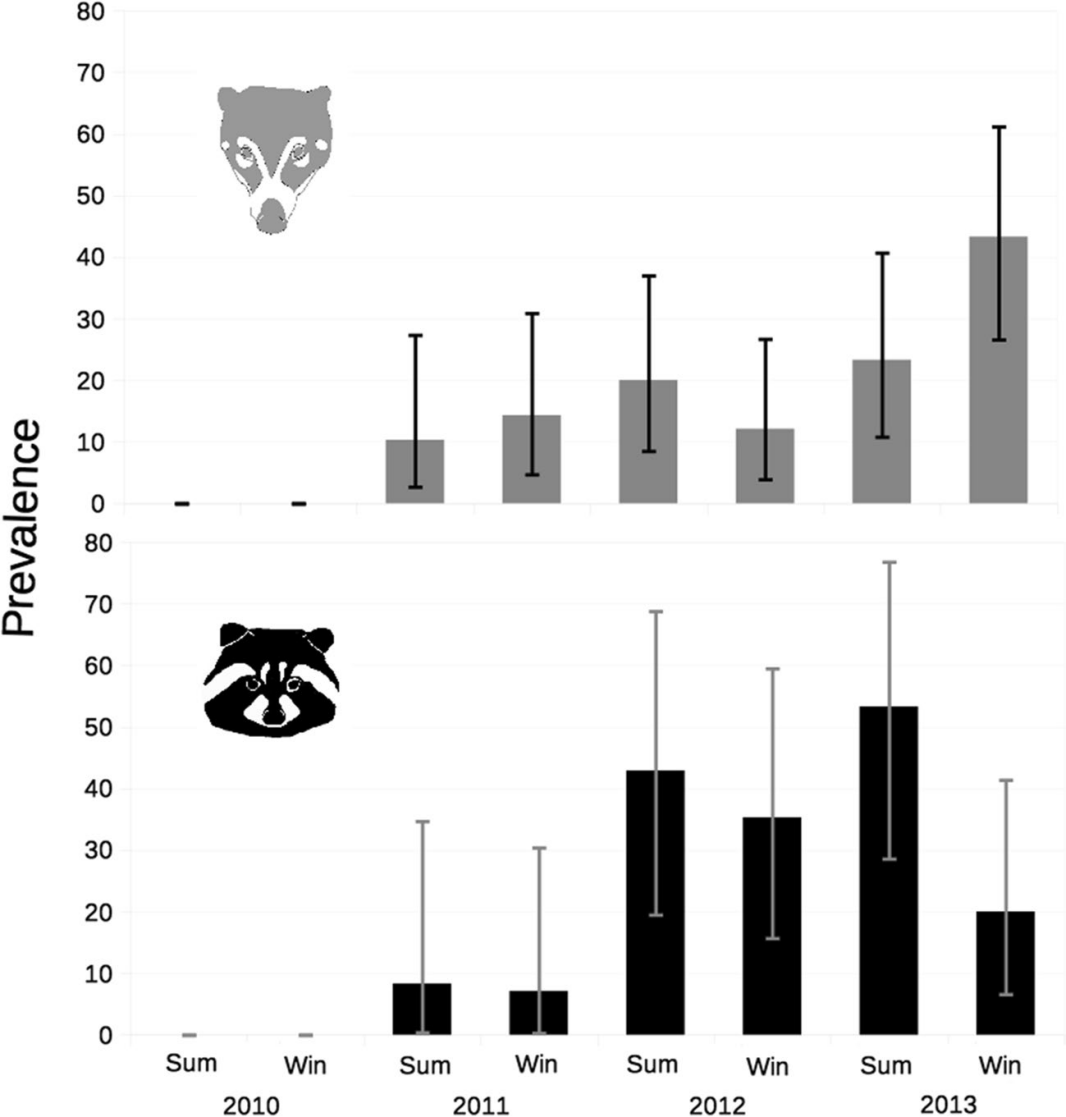
*Trichinella* seroprevalence in procyonids captured from summer of 2010 to winter of 2013. Antibodies were detected by ELISA using the excretory-secretory antigens of *Trichinella spiralis*

Seroprevalence in coatis and raccoons across time is shown in Fig. 2. For lowest seroprevalence was zero in 2009 and 2010 and the highest was 43.3% during winter of 2013 for coatis and 53.3% in summer of 2013 for raccoons. Recaptured coatis and raccoons showed change in *Trichinella*-positive status: fifteen animals seroconverted and remained positive, eight animals were positive since their first capture, and finally one seropositive coati became seronegative (supplementary Table 1 and 2).

In-House ELISA and WB are valuable tools for screening wildlife for infectious diseases, mainly because there are typically no specific tests or reagents available for most wild species (Rendón-Franco et al. 2014). However, these tests have some pitfalls such as cross-reactions. To overcome this issue, commercial ELISAs could be adapted to compare results with the in-house tests. Alternatively, additional tests can be employed to discharge cross-reactions. Coatis and raccoons from “Parque Museo de la Venta'', have been analysed for other helminths through in-house ELISA, WB and coproparasitoscopy techniques without evidence of cross-reactions among *Trichinella* and other helminths (manuscript in preparation). Furthermore, studies have shown that human antibodies targeting *Trichinella* surfaces antigens and ESP do not exhibit cross-reactivity with certain diseases, including helminthiases (De-La-Rosa et al. 1995; Tinoco-Velázquez et al. 2002).

Our data show the presence of *Trichinella* in a wild population within an urbanized area. The overall prevalence for both procyonid species was 18.2%, that is in accordance with other *Trichinella* prevalence reports for other carnivores around the world, and raccoon’s prevalence (24.5%) fit into USA reports (Smith et al. 1985; Pozio 2005; Gajadhar and Forbes 2010). However, it is worth nothing that previous studies used different diagnostic tests; nevertheless, despite the variation in techniques, the prevalence remain similar.

Differences between carnivore species are commonly reported. However, it has been more common to detected among species of different families for example Canidae *vs* Ursidae *vs* Felidae (Kojola et al. 2017). Prevalences between white-nosed coatis and raccoons were different and occur within the family, Procyonidae. Since both populations are sympatric (they share the same space and resources), then differences may be explained in terms of fine-scale foraging behavior or other biological differences, such as immune system (Rendón-Franco et al. 2019).

Unexpectedly, the prevalence of anti-*Trichinella* antibodies increased over time; from zero to reach up to 50% in raccoons. This sudden appearance of antibodies provide evidence to suggest a *Trichinella* introductory event in the procyonids. Variations in *Trichinella* prevalence across time have been studied by Hurníková and Dubinský (2009) in Slovakian red foxes (*Vulpes vulpes*), where they detected an increase in the prevalence from 4.9% in 2000 to 20.5% in 2007; they stated that fluctuations could be attributed to fox migrations from endemic regions and to the effects of anthropogenic disturbance favoring food competition, cannibalism, predation/scavenging of domestic animals and consumption of garbage*.* For both populations in Parque-Museo de la Venta park, common migratory events do not occur because the area is isolated by a surrounding urbanization. Then, because it is unlikely that migration events have occurred, the *Trichinella* appearance may be due to a predatory event of synanthropic birds or mammals that got into the park, such as invasive rodents or domestic cats (Thi et al. 2014). Scavenging could be another source of infection, since raccoons and coatis were seen scavenging by park staff. Both raccoons and coatis eat a wide variety of foods, including garbage and dead animals (Rodrigues et al. 2021), for white-nosed coatis even cannibalism has been reported (Russell 1981), that seems to be favored by overpopulation. Besides, both species were observed scavenging garbage in trash cans. Indeed, park staff reported that they hunt wild birds, howler monkeys (*Alouatta palliata*), feral cats (*Felis catus*), squirrels (*Sciurus* sp.), boa (*Boa imperator*) and porcupines (*Coendou mexicanus*) which roam free and are part of the park's local fauna.

Once *Trichinella* arrive to the park, it reaches an endemic status, as Takumi et al. (2010) explain, the *Trichinella* persist in a closed population of rats under the assumption of cannibalism. For procyonids in the park cannibalism is feasible because higher densities. After initial *Trichinella* entry into the procyonid community, the endemic state could be explained in terms of fluctuating cannibalism.

Antibodies decay against certain microorganisms has been observed in procyonids (Villalobos et al. 2020; Rendón-Franco et al. 2023), as well as in both wild (Davidson et al. 2009) and domestic animals against *Trichinella* (Hill et al. 2007). An explanation is based in the survival of *Trichinella* within the host. Once *Trichinella* dies (Hill et al. 2007) and ceases to produce E-S antigens, the production of antibodies also stops due to the lack of antigenic stimulation. Additionally, *Trichinella* induces immune modulation, including the activation of B reg and T reg cells, resulting in immunosuppression and potentially contributing to the decay of antibodies (Bruschi et al. 2022).

Based on our findings, it is evident that both *N. narica* and *P. lotor* come in contact with *Trichinella* spp. Therefore, neotropial procionids could be potentially serve as a source of *Trichinella* infections. Furthermore, it is important to explore differences between host species, to get a better understanding of the role of different carnivores in *Trichinella*´s sylvatic cycle. Finally, in future research endeavors, the inclusion of additional techniques such as artificial digestion or PCR analysis of animal tissues will be necessary to determine the specific *Trichinella* species involved.

### Supplementary Information

Below is the link to the electronic supplementary material.

Supplementary file1 (DOCX 184 KB)

## Data Availability

All relevant data is available in the manuscript or as supplemental material.
