# Supplementary material for: Search for antibodies against Trichinella in two synanthropic Procyonidae species from southeast Mexico: white-nosed coatis (Nasua narica) and raccoons (Procyon lotor)
Source: Vet Res Commun. 2023 Nov 8;48(2):1211–7. doi: 10.1007/s11259-023-10248-1 (PMC10998775; doi:10.1007/s11259-023-10248-1)
Supplement: Supplementary file 1 — Supplementary file1 (DOCX 184 KB) [file 11259_2023_10248_MOESM1_ESM.docx]

Supplementary table 1. Antibodies to *Trichinella* in serum samples of capture/recapture coatis (summer 2010 to winter 2013). Results of ELISA and WB were analyzed by season of the year. Figure shows positive (+) and negative (0) samples. Blanks mean no tested sample.

Supplementary table 2. Antibodies to *Trichinella* in serum samples of capture/recapture raccoons (summer 2010 to winter 2013). Results of ELISA and WB were analyzed by season of the year. Figure shows positive (+) and negative (0) samples. Blanks mean no tested sample.
